# Correction

**DOI:** 10.1080/20018525.2025.2539662

**Published:** 2025-08-04

**Article title**: Long-term non-invasive ventilation for COPD patients following an exacerbation with acute hypercapnic respiratory failure: a randomized controlled trial

**Authors**: Caroline Hedsund, Kasper Linde Ankjærgaard, Tine Peick Sonne, Philip Tønnesen, Ejvind Frausing Hansen, Helle Frost Andreassen, Ronan M. G. Berg, Jens-Ulrik Stæhr Jensen and Jon Torgny Wilcke

**Journal**: *European Clinical Respiratory Journal*

**Bibliometrics**: Volume 10, Number 1, pages 1-11

**DOI**: https://doi.org/10.1080/20018525.2023.2257993

The author has discovered an error related to one of the health-related quality of life measurements reported in the published article. The error occurred during the recoding of patient-reported Severe Respiratory Insufficiency Questionnaire (SRI) items for analysis.

This has impacted Tables 1 and 4. The updated tables are provided below. These corrections do not affect the description, interpretation, or original conclusions of the article. The authors apologize for any inconvenience caused.

**Table 1:** **Baseline characteristics and index admission data** for patients included in the Intention-To-Treat (ITT) analysis, presented as median (IQR) unless otherwise stated.

Baseline characteristics	n	ITT	
		LT-NIV (n=28)	Control (n=27)
Age, years,	55	70 (66-75)	73 (64-77)
Female Sex, n (%)	55	16 (57%)	13 (48%)
Body mass index (BMI), kg/m^2^	52	27.7 (21.7-32.8)	21.8 (19.1-26.6)
Long-term oxygen treatment (LTOT), n (%)	55	14 (50%)	16 (59%)
Pack years	54	46 (35-50)	50 (30-55)
Smoking history, n (%)	55		
- Current		13 (46%)	11 (41%)
- Previous		15 (54%)	15 (56%)
- Never		0 (0%)	1 (4%)
Forced expiratory volume, first second of expiration (FEV_1_), liter	54	0.6 (0.5-0.8)	0.6 (0.5-0.8)
FEV_1_, % predicted	54	24 (21-30)	28 (18-33)
Forced vital capacity (FVC), liter	54	1.4 (1.1-1.9)	1.7 (1.4-2.0)
FVC, % predicted	53	44 (41-53)	51 (43-67)
FEV_1_/ FVC ratio	54	0.4 (0.4-0.5)	0.4 (0.3-0.4)
COPD related medication, n (%)	55		
- Long-acting muscarinic antagonist (LAMA)		27 (96%)	24 (89 %)
- Long-acting beta2 agonist (LABA)		27 (96%)	27 (100%)
- Inhalation corticosteroids (ICS)		19 (68%)	23 (85%)
- Oral corticosteroids (OCS)		2 (7%)	2 (7%)
- Theophylline		4 (14%)	1 (4%)
- Roflumilast		3 (11%)	2 (7%)
- Azithromycin		3 (11%)	1 (4%)
No. Moderate AECODP last year	55	2 (1-4)	2 (0-4)
No. admissions respiratory cause last year	55	1 (0-4)	1 (0-3)
No. admissions with AHRF last year	55	0 (0-1)	0 (0-1)
≥ 2 AHRF last year, n (%)	55	13 (46%)	10 (37%)
Comorbidities n (%)	55		
- Heart failure		8 (29%)	6 (22%)
- Osteoporosis		12 (43%)	7 (26%)
- Depression		5 (18%)	5 (19%)
***Medical Research Council* dyspnoea score (MRC)**	53	4 (3-4)	4 (3-4)
Severe Respiratory Insufficiency questionnaire (SRI)	50	50.9 (38.6 – 58.3)	50.8 (44.1 – 55.8)
COPD Assessment Test (CAT)	53	22 (15-25)	20 (15-26)
Epworth sleepiness scale (ESS)	52	9 (5-11)	6 (3-10)
**Index admission data**			
Length of hospital stay, days	52	7 (5-11)	6 (4-10)
Blood gas at admission, before initiation of acute NIV			
- Partial arterial carbon dioxide pressure (PaCO_2_), kPa	55	10.0 (8.6-11.3)	9.0 (7.9-10.5)
- Partial arterial oxygen pressure (PaO_2_), kPa	55	10.2 (8.2-12.6)	9.9 (8.0-11.5)
- pH	55	7.29 (7.23-7.32)	7.28 (7.25-7.32)
- Oxygen saturation (SaO_2_) (%)	53	92 (89-95)	92 (90-95)
- Standard bicarbonate (StHCO_3_)	53	27.5 (24.3-33.3)	28.5 (25.6-31.8)
- Base Excess (BE)	52	6.0 (2.6-8.8)	5.8 (3.2-10.0)
Blood gas at discharge			
- PaCO_2_ (kPa)	50	6.9 (6.3-7.6)	6.9 (6.1-7.5)
- PaO_2_ (kPa)	49	7.9 (7.4-9.1)	8.9 (7.2-10.6)
- pH	50	7.43 (7.41-7.45)	7.42 (7.39-7.45)
- SaO_2_	49	92 (90-94)	93 (90-96)
- StHBO_3_	49	31.5 (30.1-34.2)	30.8 (28.7-32.9)
- BE	48	8.7 (6.6-12.1)	8.3 (5.4-11.4)
PaCO_2_ ≤ 6.0 kPa at discharge, n (%)	50	4 (16%)	8 (30%)
NIV maximum pressures, cmH_2_O			
- Inspiratory positive airway pressure (IPAP)	47	21 (18-24)	18 (16-22)
- Expiratory positive airway pressure (EPAP)	47	5 (5-6)	5 (5-6)

**Table 4:** **Secondary outcomes**, including change from baseline in absolute values. Forced expiratory volume within first second of expiration (FEV_1_ %), Body mass index (BMI), Medical Research Council dyspnoea score (MRC), COPD Assessment Test (CAT), Severe Respiratory Insufficiency questionnaire (SRI), Epworth sleepiness scale (ESS). PP: patients included in the per protocol analysis. Presented as median (IQR) Analysis: Wilcoxon test

	PP: 90 days follow up				PP: 365 days follow up			
	Baseline (discharge)	At 90 days	Change within 90 days	n	Baseline (discharge)	At 1 year	Change within 1 year	n
**FEV_1_ %**								
**- LT-NIV**	24 (20-32)	24 (19-45)	-1.0 (-7.0 – 2.0)	18	24 (21-34)	35 (27-55)	-3.0 (-12.5 – -0.6)	11
**- Control**	29 (20-34)	26 (19-29)	1.4 (-1.9 – 4.1)	13	29 (18-35)	23 (15-35)	-0.4 (-2.6 – 2.0)	7
**p**	0.816	0.574	0.237		0.796	0.112	0.202	
**BMI**								
**- LT-NIV**	27.8 (21.2-33.6)	27.5 (24.4-32.1)	-0.4 (-1.8 – 0.6)	18	27.8 (20.1-34.4)	30.9 (27.4-33.7)	0.1 (-2.4 – 3.0)	10
**- Control**	22.9 (20.0-27.2)	24.1 (19.8-27.0)	-0.1 (-1.1 – 0.9)	13	21.8 (18.8-26.6)	26.7 (21.5-28.6)	-1.3 (-2.4 – 1.7)	7
**p**	0.113	0.051	0.275		0.128	0.043	0.601	
**MRC**								
**- LT-NIV**	4 (3-5)	3 (3-4)	1(0-1)	19	4 (3-5)	3 (3-5)	0 (0 – 1)	13
**- Control**	4 (3-4)	3 (3-4)	1(0-1)	13	4 (3-4)	3 (3-4)	0 (-1 – 1)	7
**p**	0.523	0.391	0.647		0.148	0.881	0.901	
**CAT**								
**- LT-NIV**	22 (16-25)	19 (10-27)	2(-1 – 7)	19	22 (16-25)	15 (12-22)	2 (-3 – 8)	11
**- Control**	20 (16-26)	18 (15-20)	4 (-2 - 8)	13	23 (20-27)	20 (16-28)	4 (-8 – 8)	7
**p**	0.874	0.872	0.711		0.561	0.205	0.893	
**SRI**								
**- LT-NIV**	50.4 (36.4 – 59.1)	58.6 (34.0 – 64.2)	-3.1 (-14.5 – 5.8)	19	51.1 (38.9 – 59.7)	59.9 (50.4 – 71.9)	-7.2 (-13.6 – 2.0)	11
**- Control**	50.9 (43.8 – 56.4)	48.0 (45.8 – 69.1)	-5.3 (-14.6 – 2.8)	12	51.9 (45.2 – 56.5)	56.2 (42.5 – 59.6)	-1.0 (-5.1 – 6.6)	7
**p**	0.264	0.904	0.369		0.179	0.424	0.456	
**ESS**								
**- LT-NIV**	8 (4-11)	4 (2-9)	2 (-3 – 6)	19	8 (5-11)	6 (1-9)	0 (-1 – 6)	11
**- Control**	7 (3-9)	3 (2-6)	1 (-1 – 3)	13	5 (4-9)	2 (2-8)	2 (0-4)	7
**p**	0.351	0.730	0.696		0.221	0.561	0.828	

